# What is the best way to keep the patient warm during technical rescue? Results from two prospective randomised controlled studies with healthy volunteers

**DOI:** 10.1186/s12873-023-00850-6

**Published:** 2023-08-04

**Authors:** Martin Lier, Christopher Jebens, Annette Lorey-Tews, Tim Heyne, Nils Kunze-Szikszay, Johannes Wieditz, Anselm Bräuer

**Affiliations:** 1 Department of Anesthesiology, University Medical Center Göttingen, Robert-Koch-Strasse 40, 37075 Göttingen, Germany; 2 Department of Anesthesiology, Intensive care, Emergency and Pain medicine, Asklepios Clinic Altona, Paul-Ehrlich-Strasse 1, 22763 Hamburg, Germany; 3 Department of Anesthesiology and Intensive care medicine, Buchholz Hospital, Steinbecker Strasse 44, 21244 Buchholz in der Nordheide, Germany; 4 Department of Medical Statistics, University Medical Center Göttingen, Humboldtallee 32, 37073 Göttingen, Germany

**Keywords:** Accidental hypothermia, Technical rescue, Preclinical warming, Warming methods, Forced air warming, Halogen floodlight, Blanket

## Abstract

**Background:**

Accidental hypothermia is a manifest problem during the rescue of entrapped victims and results in different subsequent problems as coagulopathy and wound infection. Different warming methods are available for the preclinicial use. However, their effectiveness has hardly been evaluated.

**Methods:**

In a first step a survey among German fire brigades was performed with questions about the most used warming methods. In a second step two crossover studies were conducted. In each study two different warming method were compared with forced air warming – which is the most frequently used and highly effective warming method in operation rooms (Study A: halogen floodlight vs. forced air warming; Study B: forced air warming vs. fleece blanket). In both studies healthy volunteers (Study A: 30 volunteers, Study B: 32 volunteers) were sitting 60 min in a cold store. In the first 21 min there was no subject warming. Afterwards the different warming methods were initiated. Every 3 min parameters like skin temperature, core body temperature and cold perception on a 10-point numeric rating scale were recorded. Linear mixed models were fitted for each parameter to check for differences in temperature trajectories and cold perception with regard to the different warming methods.

**Results:**

One hundred fifty-one German fire brigades responded to the survey. The most frequently used warming methods were different rescue blankets (gold/silver, wool) and work light (halogen floodlights). Both studies (A and B) showed significantly (p < 0.05) higher values in mean skin temperature, mean body temperature and total body heat for the forced air warming methods compared to halogen floodlight respectively fleece blanket shortly after warming initiation. In contrast, values for the cold perception were significantly lower (less unpleasant cold perception) during the phase the forced air warming methods were used, compared to the fleece blanket or the halogen floodlight was used.

**Conclusion:**

Forced air warming methods used under the standardised experimental setting are an effective method to keep patients warm during technical rescue. Halogen floodlight has an insufficient effect on the patient’s heat preservation. In healthy subjects, fleece blankets will stop heat loss but will not correct heat that has already been lost.

**Trial registration:**

The studies were registered retrospectively on 14/02/2022 on the German Clinical Trials registry (DRKS) with the number DRKS00028079.

**Supplementary Information:**

The online version contains supplementary material available at 10.1186/s12873-023-00850-6.

## Background

Accidental hypothermia as a consequence of severe trauma is regularly observed [1–5]. Severe hypothermia occurs as a result of the trauma induced changes in thermoregulation, hypovolemia and shock [6], sympathetic activation, delivery of unheated infusions [6], anaesthesia [7], undressing and performing invasive measures.

Entrapped victims after traffic accidents represent a special risk group, especially in winter, as they are usually seriously injured and exposed to the cold for a long time until they are ready for transport in the ambulance. In the recent decades this risk has become even greater, as the changed design of modern cars has led to a significant increase in protracted technical rescue [8, 9].

Unfortunately, core body temperature is rarely measured in prehospital settings. In a study by Eidstuen et al. [10], it was shown that 73% of patients are already hypothermic when the rescue team arrives. Thereafter, changes in core body temperature tend to be smaller during transport and shock room care [10]. As a result, most trauma patients arrive hypothermic at the emergency department [11].

Hypothermia affects both the plasmatic and cellular components of coagulation and is a major contributor to coagulopathy in these patients [12, 13]. At the same time, there is increased fibrinolytic activity [13]. But hypothermia also has a decisive influence on the immune system in severely injured patients. It has been shown that patients who suffer from hypothermia during the primary phase of trauma care have a significantly higher incidence of wound infection, pneumonia and even sepsis [4, 14].

Whether accidental hypothermia after severe trauma is an independent factor for increased mortality or only a surrogate parameter of the severity of the injury and haemorrhage is controversially discussed in the literature. On the one hand, many authors were able to identify hypothermia as an independent mortality factor in their studies [3, 5, 15–17]. In contrast, other authors were unable to identify an independent relationship between hypothermia and increased mortality [2, 4, 18]. Irrespective of this discussion, however, the restoration or maintenance of normothermia is clearly recommended for trauma patients [19].

The following studies were designed to address three questions related to the care of entrapped trauma victims.

1. What is the most frequently used method for heat protection for entrapped trauma victims carried out by the fire brigades in Germany?
2. Are these thermal protection methods effective?
3. Could the pre-hospital use of forced air warming be a viable alternative?

## Methods

To answer the first question, a representative survey was conducted in Germany. From a list of all German municipal authorities 300 fire brigades were randomly selected and contacted. For this survey a questionnaire was developed. Beside other questions these fire brigades were asked by which means they usually protect entrapped patients after car accidents from hypothermia (For the complete questionnaire please see the supplementary material - supplement 1).

In the next part of the work, the most frequently used heat protection procedures used were then examined in volunteers and compared to forced air warming, which is a frequently used and highly effective warming methods in operation rooms [20]. This was done in two studies, both of which had been approved by the local Ethics Committee of the Medical Faculty of the Georg-August University Göttingen with the number 6/8/11 and the studies were registered retrospectively on 14/02/2022 on the German Clinical Trials registry (DRKS) with the number DRKS00028079.

The same methodology was used in both studies except for the different warming methods. Both studies were performed in a crossover fashion. We planned to restrict enrolment to 30 participants in each study due to economic reasons.

In the first study (Study A), two active heat protection methods were compared. The first method was the usage of a halogen floodlight as used frequently in preclinical use (Flutlichtstrahler BS 1000, Karl Meister GmbH Feuerwehrtechnik, Reutlingen, Germany). This floodlight is used by many fire brigades in Germany and has a power of 1000 W according to the manufacturer. The floodlight was mounted on a tripod at a height of 1 m and placed at a distance of 2 m from the centre of the volunteer’s body at a right angle to it. The other method used was a forced air warmer intended for preclinical use (Polarn 4000, Eberspächer, Esslingen, Germany). This forced air warmer is operated with diesel fuel and has 4 heating stages with a heat flow between 900 and 4000 watts according to the manufacturer’s specifications. During the test series, only heating level 2 (= 2000 watts) was used. The generated warm air was then transported to the patient via a 1.5 m long and 90 mm wide air hose. A commercially available plastic foil with dimensions of 2 × 2.5 m was used as a warming blanket. The volunteers were covered with the foil and the warm air hose was passed under the foil at the subject’s feet.

**Fig. 1:**
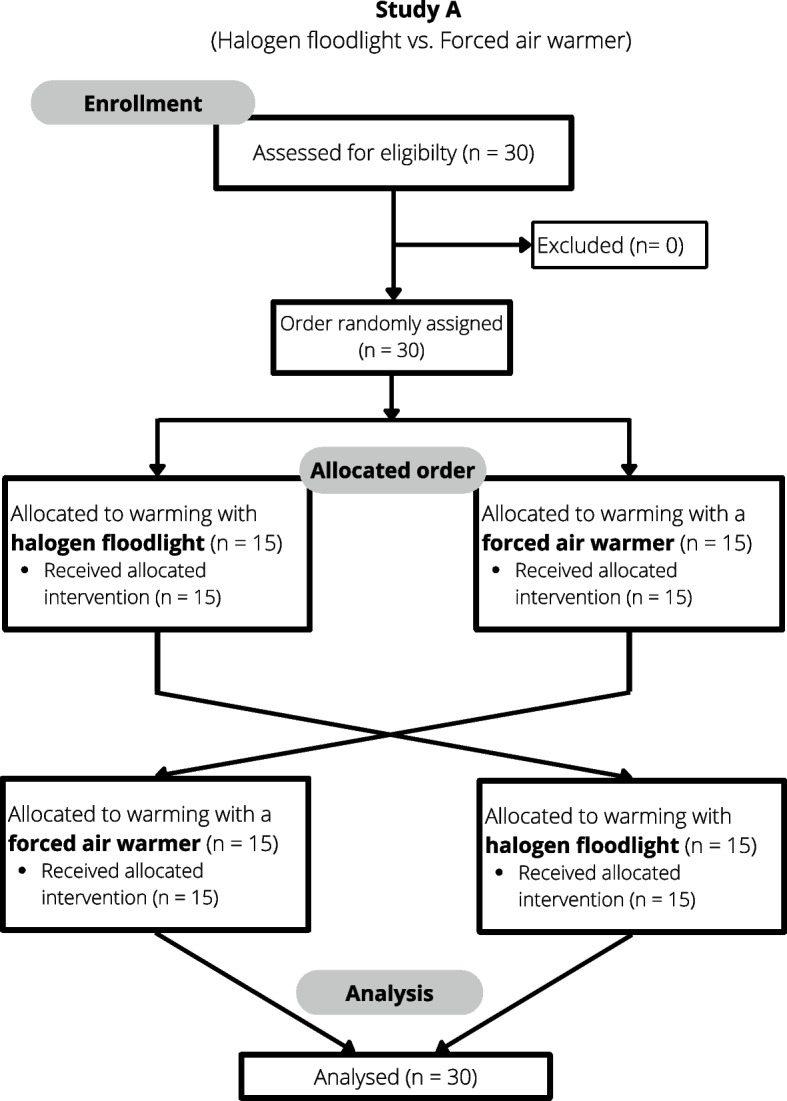
CONSORT flow chart of volunteer study A

For study A, 30 subjects were recruited and included after informed and written consent (Fig. 1).

In the second study (Study B), a passive and an active heat protection method were compared. As a passive method, a disposable fleece blanket as used in prehospital care (Reintex GmbH, Ketsch, Germany) was used. According to the manufacturer, the chosen blanket is produced for use on patients in rescue services, fire brigades and ambulance transport. It is a disposable blanket with a cover made of a non-woven polypropylene fabric and a polyester wool filling. The size is 1.90 m x 1.10 m. The weight of the blanket is given by the manufacturer as approx. 250 g. The active method used was a clinical used forced air warmer (WarmTouch WT 5900, Nellcor Tyco Healthcare Group LP, Pleasanton, USA) with CareQuilt adult full body blanket. The forced air warmer generates an air flow with a constant temperature of 43 °C. The experimental procedure for the volunteers was subsequently identical.

**Fig. 2:**
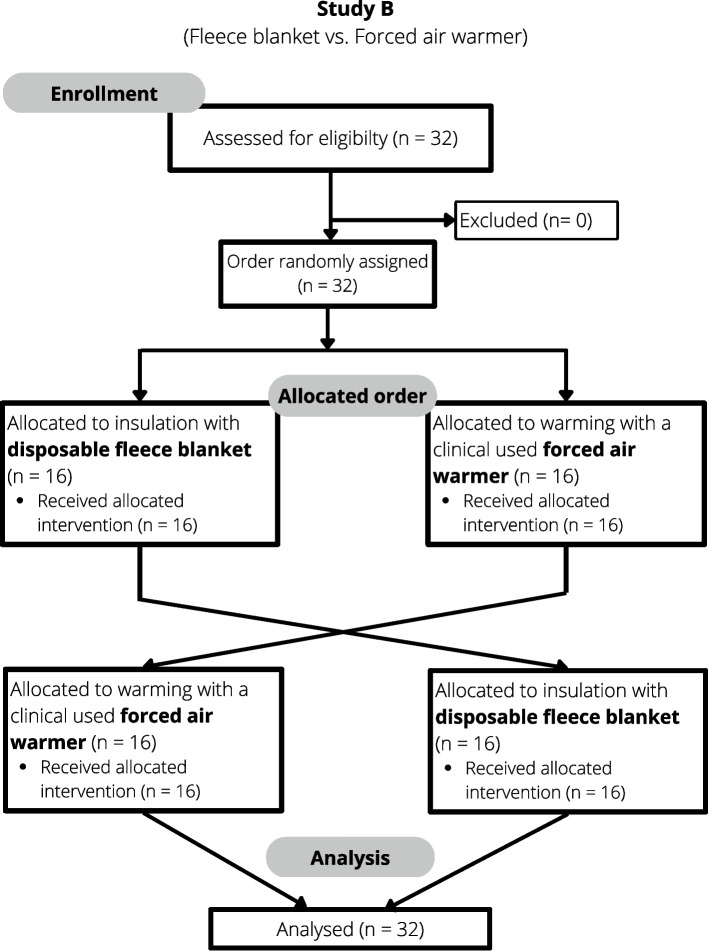
CONSORT flow chart of volunteer study B

For the second study (study B), 32 subjects were recruited and included in the study after informed and written consent (Fig. 2).

Participating volunteers of both studies had to meet the following inclusion criteria:

- Able to understand written informed consent
- Age ≥18 years, ≤ 65 years
- ASA I-II
- Body mass index < 30 kg/m²
- Subjective well-being at the time of the examination

Exclusion criteria were defined as:

- Upper respiratory tract infection
- Existing pregnancy
- Regular medication

The following criteria were defined as termination criteria for each experiment:

- Core body temperature below 34 °C
- Heart rate above 120/min
- Heart rate below 50/min
- Decrease of SpO_2_ to values below 95%
- Pathological ECG changes
- Subjective malaise
- Subject’s wish to stop the test

### Experimental design and conduct

Each study (A and B) was designed in a crossover fashion with a washout period of at least 24 h between the two interventions. Volunteers received both warming methods (interventions) each. The order of the warming methods was randomised by letting the participants drawing lots.

Only some of the volunteers where the same in study A and study B.

In order to simulate the environmental situation of a traffic accident in a standardised way, a driver’s seat of a passenger car was placed in a 7500 m² cold store at a stable ambient temperature of 3 °C ± 1 °C. The volunteers were placed in the driver’s seat of a passenger car dressed for the test period in a T-shirt, shorts, underwear, stockings and shoes. They were asked to move as little as possible during the experiment.

Monitoring was applied before the start of the experiments: ECG, oscillometric blood pressure measurement and peripheral oxygen saturation. Additionally, core body temperature was measured by means of a tympanic contact temperature probe (Tympanic Temperature Probe with Foam, Disposable, 400 Series, GE Healthcare GmbH, Solingen, Germany). This measuring probe was placed in the right auditory canal and pushed forward until the volunteers noticed contact with the eardrum. The ear canal was then covered with cotton wool and a bandage was applied around the head to secure it.

These parameters were then continuously monitored and documented using a Propaq EL 104 E monitor (Welch Allyn Limited, Navan, Ireland). In addition, skin temperature was determined every 3 min using the KIRAY 300 infrared thermometer (ELECTRO-MATION GmbH, Hamburg, Germany) at the following skin sites:

- Upper arm (medial outside right).
- Chest (medial approximately 5 cm above the mammillary line).
- Thigh (medial outer right).
- Calf (medial right).

Occurring muscle tremors were recorded according to the Bedside Shivering Assessment Scale (BSA) (Table 1) [21].

**Table 1:** Bedside shivering assessment scale [21]

Score	Type of shivering	Location
0	None	No shivering is detected on palpation of the masseter, neck, or chest muscle
1	Mild	Shivering localised to the neck or thorax only
2	Moderate	Shivering involves gross movement of the upper extremities (in addition to neck and thorax)
3	Severe	Shivering involves gross movements of the trunk and upper and lower extremities

Furthermore, the subjective cold perception was determined by means of a numerical rating scale (NRS). To this end, volunteers were asked to describe their respective cold perception on a numeric scale from 0 (pleasant feeling of warmth) to 10 (maximum feeling of cold).

In addition, the volunteers were asked about their subjective evaluation of the heating method. The following answers were available for selection:

The heating method:

- Did not have any effect at all
- Warmed only slightly
- Warmed moderately
- Warmed considerably

For the experiment itself, volunteers were placed on the car seat and the first measurement was started at time 0 min. The experimental set-up was intended to reflect the typical time course of prehospital care. With an average duration of the prehospital time between accident and hospital admission for severely injured patients of roughly 60 min (Trauma Register of the DGU Annual Report 2021, https://www.traumaregister-dgu.de/fileadmin/user_upload/TR-DGU_Jahresbericht_2021.pdf), it was assumed that it takes approx. 21 min for rescue to begin after the accident, with rescue of the entrapped victim taking an average of additional 39 min. Therefore, the subjects were only exposed to the cold for the first 21 min and then heat protection was carried out for 39 min. Subsequently, the experiment was terminated.

### Data analysis

The following data were used or calculated for the statistical evaluation of the temperature readings:

- Core body temperature (CBT)
- Mean skin temperature (MST) according to the formula of Ramanathan [19]:

$$\mathrm{MST}\;=\;0.3\;\cdot\;\left({\mathrm T}_{\mathrm{chest}}\;+\;{\mathrm T}_{\mathrm{Upper}\;\mathrm{arm}}\right)\;+\;0.2\;\cdot\;\left({\mathrm T}_{\mathrm{Thigh}}+\;{\mathrm T}_{\mathrm{Calf}}\right)$$

- Mean body temperature (MBT) [22]

$$\mathrm{MBT}\;=\;0.64\;\cdot\;\mathrm{CBT}\;+\;0.36\;\cdot\;\mathrm{MST}$$

- Total body heat (TBH) according to Burton [23]

$$TBH\;=\;\left(0.64\;\cdot\;CBT\;+\;0.36\;\cdot\;MST\right)\;\cdot\;weight\;(in\; kg)\;\cdot\;\left(3.475\;kJ\;\cdot\;kg^{-1}\;\cdot\;^\circ C^{-1}\right)$$

where the term in the last parentheses is the specific heat capacity of human body tissu

- Bedside Shivering Assessment Scale
- Subjective perception of cold on a numeric rating scale (NRS Score)
- Subjective evaluation of the heating method

### Statistical analysis

Since this study aimed at a qualitative comparison of different warming methods, no sample size computation was performed. We restricted the number of participants per study to 30 in study A and 32 patients in study B due to economic reasons.

Descriptive statistics are reported as numbers and frequencies or median [1st; 3rd quartile] as appropriate. If not stated otherwise, tests were performed two-sided on a significance level of 5%. Parameter estimates are provided with corresponding 95% confidence intervals (95%-CI).

For the course of all measured temperatures and temperature sensitivity scores over time we fitted linear mixed effects models with temperature (or the score) as dependent variable, time and warming method as fixed effects with corresponding in-between interaction and random subject effects to account for repeated measurements. All pairwise contrast tests have been conducted on the fit models to test for differences between warming methods at each time point. Resulting p-values have been corrected for multiple testing using Tukey adjustment. Data were analyzed using R version 4.2.2 (The R Foundation for Statistical Computing, Vienna, Austria).

## Results

Of the 300 fire brigades contacted, 151 (50.3%) responded. One of the most frequently used warming methods was the use of different blankets. Halogene floodlight was another frequently mentioned method to keep the patient warm – alone or in combination with blankets.

### Study A

Baseline demographics of the subjects of study A are shown in Table 2.

**Table 2:** Demographic data of the test persons of volunteer study A

**Sex**, male/female	21/9
**Height**, Median in cm (25%/75% quantiles)	181 (170/186)
**Weight**, Median in kg (25%/75% quantiles)	80 (73/89)
**BMI**, Median in kg/m² (25%/75% quantiles)	24.9 (22.2/27)

### Core body temperature, mean skin temperature and mean body temperature

**Fig. 3:**
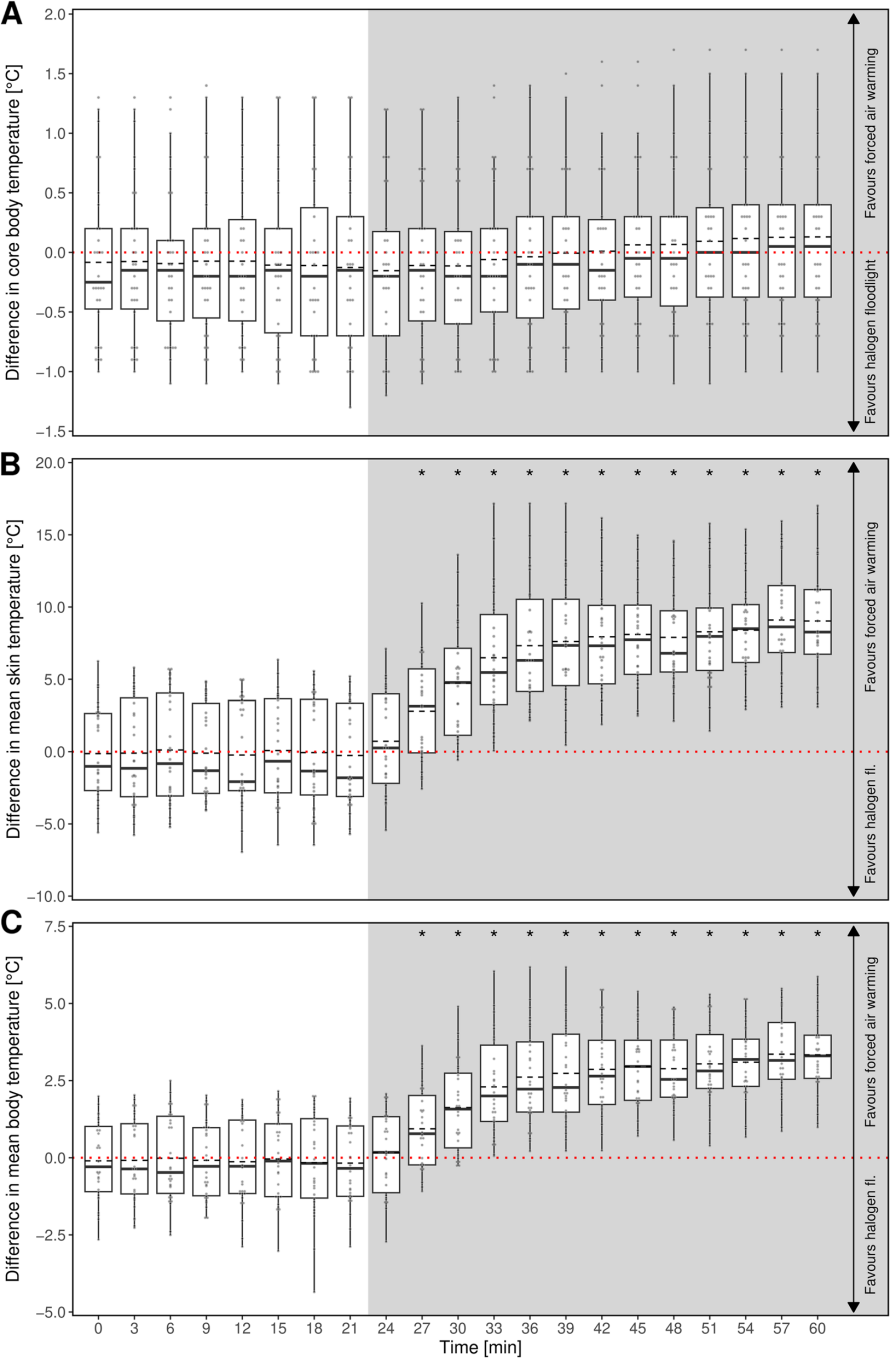
Intraindividual differences in (**A**) core body temperature, (**B**) mean skin temperature and (**C**) mean body temperature between treatment with forced air warming and halogen floodlight. The distribution of the temperature differences for a given time are displayed as boxplots with additional means (dashed lines). Positive values indicate a higher temperature with forced air warming. The dotted red line indicates indifference between the two methods. Individual data points as grey dots. The grey shaded region shows the time period of warming intervention. Asterisks indicate time points of statistically significant differences between methods

For study A, all measured temperatures (mean skin and core body temperature) showed a wide range between the participants at the same time point and the same warming method. With regard to each participant at a specific time point, the core body temperature did not differ significantly neither before nor after the induction of the two different warming methods (Fig. 3A).

In contrast, the mean skin temperature of the test person during the use of the forced air warming was significantly higher - compared to the halogen floodlight (Fig. 3B).

These substantially higher values for the mean skin temperature resulted in higher values for the calculated factor mean body temperature - although the core body temperature as part of the calculation of the mean body temperature did not differ between both warming methods (Fig. 3C).

### Total body heat, bedside shivering-assessment scale and subjective perception of cold

**Fig. 4:**
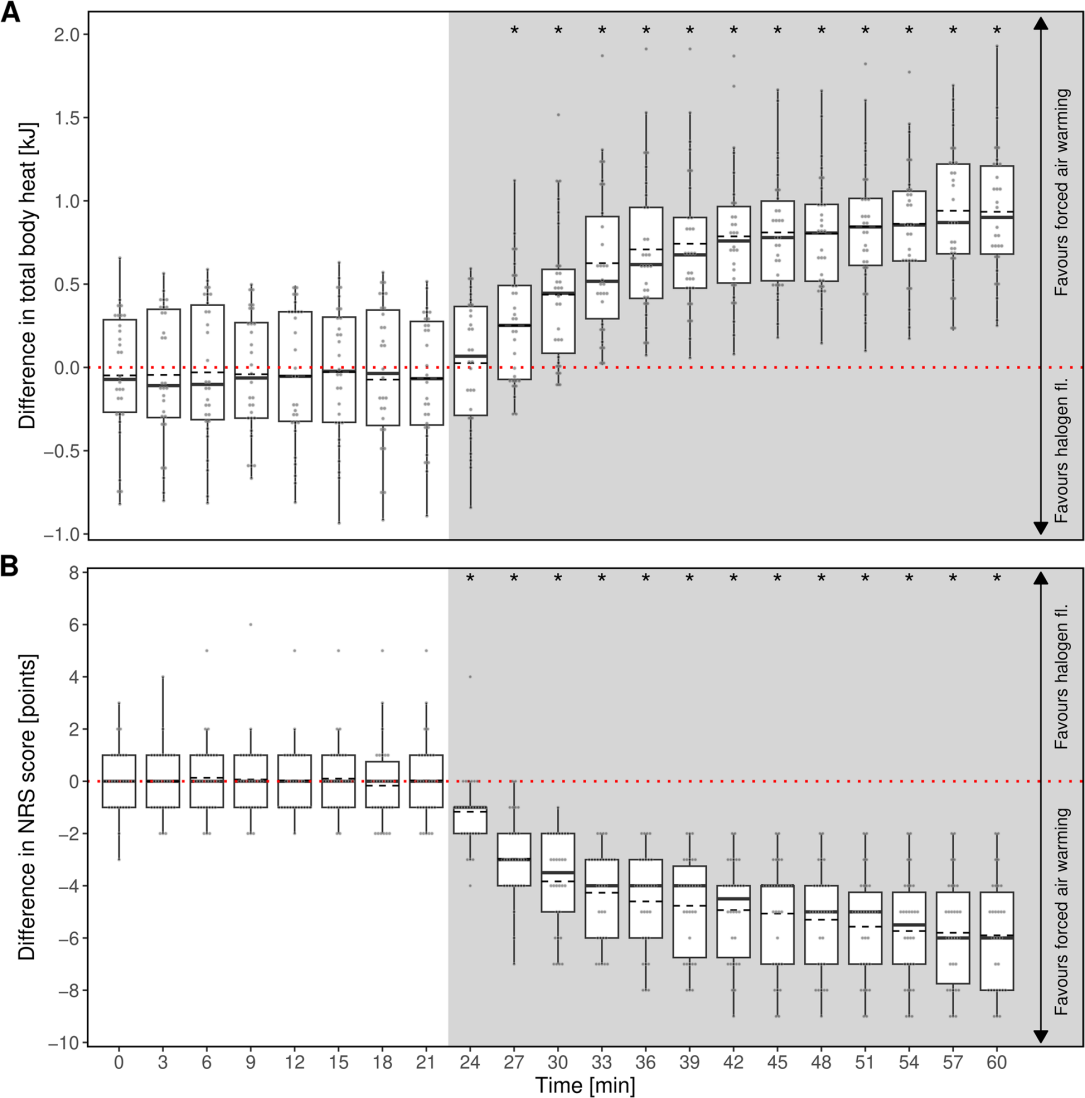
Intraindividual differences in (**A**) total body heat and (**B**) NRS score between treatment with forced air warming and halogen floodlight. The distribution of heat and NRS score, respectively, for a given time are displayed as boxplots with additional means (dashed lines). Positive values indicate higher values with forced air warming. The dotted red line indicates indifference between the two methods. Individual data points as grey dots. The grey shaded region shows the time period of warming intervention. Asterisks indicate time points of statistically significant differences between methods

The calculated parameter total body heat was significantly higher when using forced air warming compared to halogen floodlight warming shortly after warming was initiated (Fig. 4A).

Shivering was observed in three volunteers. In two volunteers, shivering only occurred during the halogen floodlight test. In one volunteer, shivering was observed during both - the halogen floodlight and the forced air warming test - but the shivering disappeared during the forced air warming test.

As a subjective parameter, the cold perception of each participant was assessed via NRS at each point in time during the trial. The participants felt less cold while being warmed with the forced air warming compared to being warmed by halogen floodlights. These differences were significant (Fig. 4B).

### Subjective evaluation of the warming method

The subjective evaluation of the heating methods yielded the following results (Table 3):

**Table 3:** Subjective assessment of the heating methods halogen radiator and forced air warmer

Assessment	Halogen floodlight (*n* = 30)	Forced air warmer (*n* = 30)
Warmed considerably	0%	100%
Warmed moderately	0%	0%
Warmed only slightly	40%	0%
Did not have any effect at all	60%	0%

Some of the test persons found the temperature of the air at the outlet of the hose of the forced air warmer to be too hot (number not documented). Subsequent measurements of the temperatures of the outflowing air showed temperatures above 60 °C (maximum 70.5 °C). The test persons responded by pulling their feet away from the entry point of the hose under the plastic foil in good time. Thermal damage to the skin or other injuries were therefore not observed.

### Study B

Demographic data of the subjects are shown in Table 4.

**Table 4:** Demographic data of the subjects of the second volunteer study

**Sex**, male/female	18/14
**Height**, Median in cm (25%/75% quantiles)	179 (170/189)
**Weight**, Median in kg (25%/75% quantiles)	79 (66/91)
**BMI**, Median in kg/m² (25%/75% quantiles)	25.1 (22.1/27.1)

### Core body temperature, mean skin temperature and mean body temperature

**Fig. 5:**
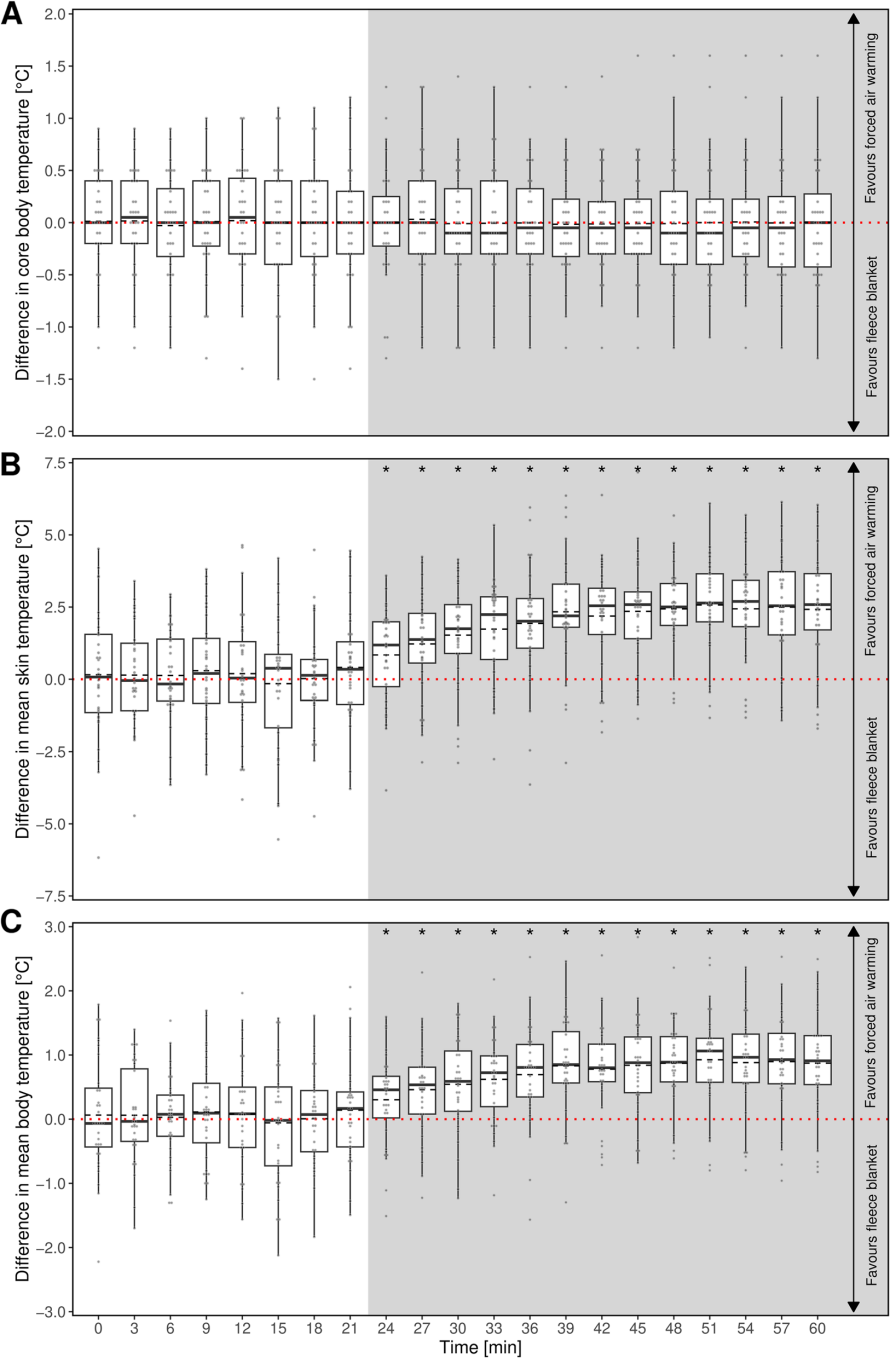
Intraindividual differences in (**A**) core body temperature, (**B**) mean skin temperature and (**C**) mean body temperature between treatment with forced air warming and warming using a fleece blanket. The distribution of the temperature differences for a given time are displayed as boxplots with additional means (dashed lines). Positive values indicate a higher temperature with forced air warming. The dotted red line indicates indifference between the two methods. Individual data points as grey dots. The grey shaded region shows the time period of warming intervention. Asterisks indicate time points of statistically significant differences between methods

Similarly to study A, the intraindividual measured temperatures (skin and core body temperature) in study B showed a wide range. During both interventions (fleece blanket and forced air-warming) the curve of the core body temperature showed the same trajectory with no significant differences (Fig. 5A). Again, in contrast to that, the mean skin temperature showed significant differences in both warming methods almost immediately after initiation of warming, with warmer mean skin temperature while using the forced air-warming (Fig. 5B).

These distinct differences for mean skin temperature resulted in a significant warmer mean body temperatures during the phase the forced air warming was used instead of the fleece blanket (Fig. 5C).

### Total body heat, bedside shivering-assessment scale and subjective perception of cold

**Fig. 6:**
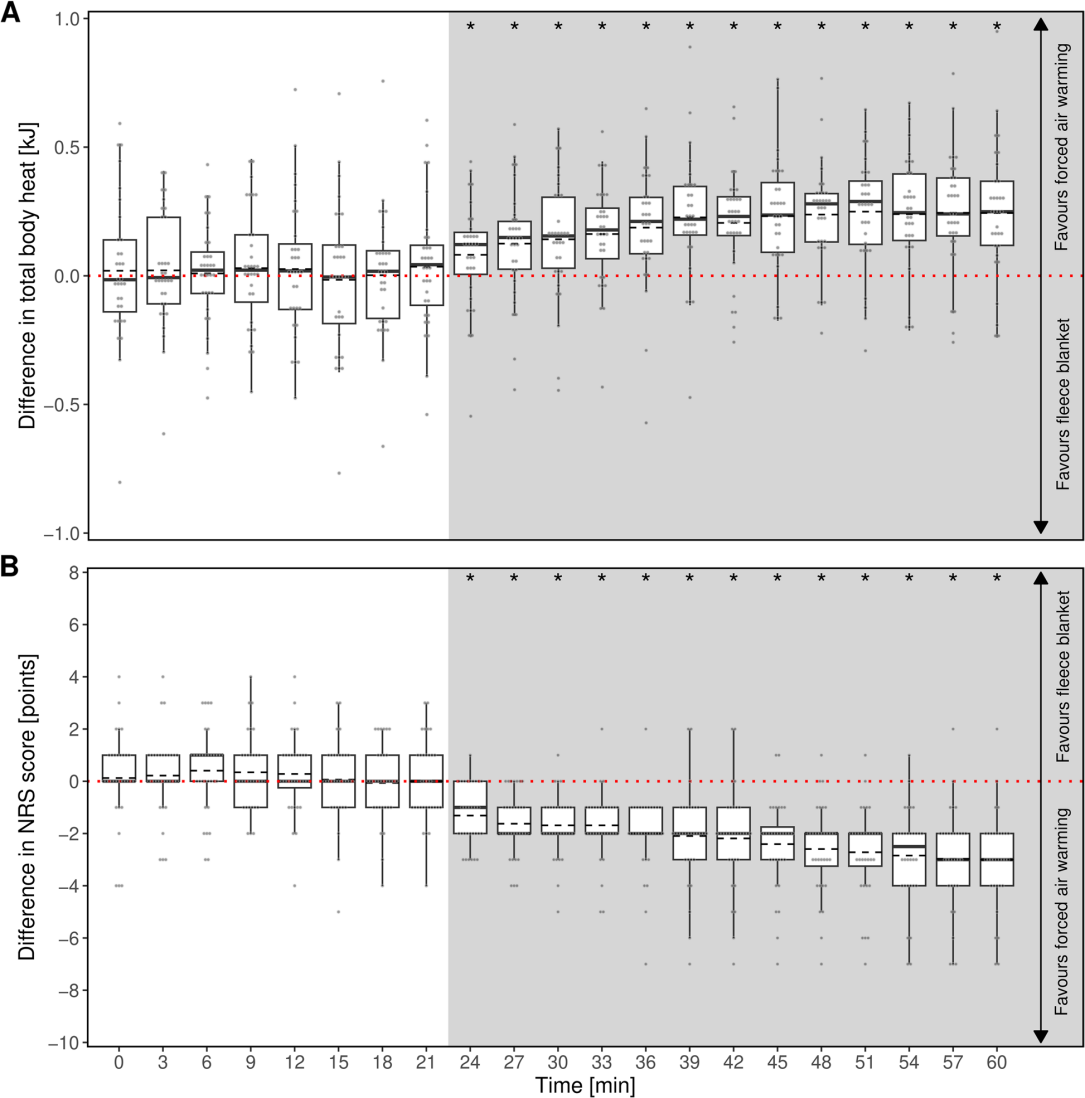
Intraindividual differences in (**A**) total body heat and (**B**) NRS score between treatment with forced air warming and warming using a fleece blanket. The distribution of heat and NRS score, respectively, for a given time are displayed as boxplots with additional means (dashed lines). Positive values indicate higher values with forced air warming. The dotted red line indicates indifference between the two methods. Individual data points as grey dots. The grey shaded region shows the time period of warming intervention. Asterisks indicate time points of statistically significant differences between methods

Until the beginning of the intervention after in minute 21 each participant showed no significant difference between the two sessions in the calculated total body heat (Fig. 6A). From this time point on, there were significantly higher values for the total body heat in favour of forced air-warming. Additionally, as a subjective parameter study participants stated a higher perception of cold (assessed via NRS) while fleece blankets were used (Fig. 6B).

Shivering was observed in four volunteers (BSA value 2). In two volunteers who were treated with the fleece blanket, there was continuous shivering, while in two volunteers who were treated with forced air warming, shivering disappeared over time.

### Subjective evaluation of the warming method

The subjective evaluation of the two methods yielded the following results (Table 5):

**Table 5:** Subjective assessment of the two methods fleece blanket and forced air warmer

Assessment	Fleece blanket (*n* = 32)	Forced air warmer (*n* = 32)
Warmed considerably	15.6%	90.6%
Warmed moderately	34.4%	9.4%
Warmed only slightly	50%	0%
Did not have any effect at all	0%	0%

A detailed presentation of the statistical results can be found in the supplementary material (supplement 2).

## Discussion

The survey responses of 151 fire brigades did show that if patients are protected, this is mainly done with passive heat protection methods like rescue blankets (gold/silver) 70% or woollen blankets 64%. This is remarkable insofar as it is known that passive heat protection methods are practically always inferior to active warming systems, as they cannot actively supply heat, but only partially conserve the remaining heat. For the frequently used rescue foils made of reflective material, there is little robust data to prove effectiveness [24–28]. Whether blankets as passive heat protection are also more frequently used because of direct protection against broken glass during the technical rescue was not specified in the questionnaire.

The most commonly mentioned active warming system in the survey was halogen floodlight (halogen floodlight only in 41% and halogen floodlight combined with rescue blankets in 39% of all cases). Scientific evidence for their effectiveness is not available. Remarkably, active forced air warming systems have not yet been used prehospital even though the idea of using forced air warming on the scene was already published in 1998 with good arguments [29]. Nevertheless, also in Portugal [30], Norway [31] or the UK [32] the use of forced air warming in emergency medicine is not widespread. The use of self-heating blankets activated by oxygen is discussed as an alternative method [33–35]. However, there are observations that these can sometimes become extremely hot [33]. Another method discussed is the use of electric carbon fibre blankets [27].

The first volunteer trial A therefore investigated whether halogen floodlights were effective and compared them with a forced air heating system approved for these purposes. The results were clear. In the subjective evaluation of the heating methods, 40% of the volunteers stated that the halogen floodlight had provided only slight heating and 60% of the volunteers stated that the halogen floodlight had done nothing at all. In contrast, all volunteers stated that the forced air warming system had provided substantial warming – a finding which was suggested by another volunteer trial by Stroop et al. [36].

Additionally, the subjective perception of cold during the trial measured by means of the 0 to 10 NRS scale was significantly lower for subjects receiving forced air warming. This subjective assessment of substantially more heat transfer of forced air warming is confirmed by a significantly higher mean skin temperature and total body heat compared to the halogen floodlight.

However, measurements of the air temperature of the forced air warming system showed values of over 60 °C. The highest temperature measured was even 70.5 °C. This was frequently answered by the volunteers by pulling their foot away from the entry point of the hose under the plastic foil. Thermal damage to the skin or other injuries were therefore not observed. However, such high air temperatures would be a risk for severe burns in unconscious people or persons with restricted movement [37–39]. With a greater distance between the nozzle of the blower and the volunteers or patients, this risk would certainly be lower, but this would also reduce the effectiveness. Due to this risk of burns, the method used here cannot be recommended in this form for safety reasons.

In the second volunteer trial B, it was therefore examined whether a forced air warming system approved in the clinic is also effective and how this compares to effective insulation using a fleece blanket.

Here, too, the results were clear. 15.6% of the volunteers felt clearly warmed by the fleece blanket, whereas this was the case in 90.6% of the cases with the forced air warming system. Likewise, in trial A the cold perception was significantly lower for the forced air warming. This subjective assessment of the heat protection methods is also supported by objective data. Both methods resulted in an increase in mean skin temperature and total body heat. However, the values were significantly higher for the forced air warming system.

Even though the comparison of the data between the two studies can only be made with great caution, as they were two separate studies with different volunteers, the results can be take as an indicator that the insulation with a fleece blanket might be better than the use of a halogen floodlight. However, the use of forced air warming seems to be more effective than insulation. As an additional factor, halogen floodlights are increasingly replaced by LED floodlights, which do not produce relevant warming radiation. As a reaction to this, some infrared devices explicitly for heating are available (i.e. Hypothermsave ®), but rarely used on scene.

The comparison of the data collected here with the literature is complicated due to very little data available. In a study by Thomassen et al. [40], three different heat protection methods were tested on volunteers in the laboratory. One of them was a disposable blanket, which showed some effectiveness, but was inferior to active heat application using a Hibbler pack. In another subject study by Hurrie et al. [41], different reheating methods were investigated. The greatest heat gain was seen with forced air warming. The same was demonstrated by Dvir et al. [42] in a torso model over a period of 480 min. The current study showed that already 6 min after the start of using warming methods there is a significant difference in mean skin temperature, total body heat and in cold perception between the different heating methods. However, when using forced air warming not only the supply of heat is crucial but also the coverage of all body parts [20]. In any case, active warming appears to be superior to passive methods [43]. Because of its higher requirement in material resources, active warming during the technical rescue has to be undertaken by the fire brigade and remains a crucial task for them. Arguments that active rewarming by preclinically used external warming methods is dangerous in hypothermic patients were weakened in a systematic review by Mydske and Thomassen [44] - although these authors also point out the poor scientific basis.

### Strengths, weaknesses and limitations of the study

Conducting randomised trials in prehospital emergency medicine is difficult and often limited by patients’ inability to give consent and high dynamics. Therefore, retrospective studies are often used. An alternative can be simulations under standardised conditions, as in the present case. The studies described here are randomised, experimental, prospective studies with healthy volunteers. In the present studies, all procedures of the experiments were standardised and strictly adhered to. Due to the crossover design of the two studies, a high statistical comparability could be achieved with a relatively small number of volunteers.

Another strength of the studies is the simultaneous assessment of objective parameters such as the change in total body heat and subjective assessments of the awake subjects. However, such a study design with volunteers requires that the subjects could not be blinded. Moreover, only the potential effects of the warming methods can be presented without showing that this is also feasible and will take place in reality. Furthermore, laboratory conditions differ from the real conditions at an accident site, as there environmental conditions such as wind strength, humidity, weather conditions such as rain or snow are not standardised. The clothing chosen for the test persons also was less insulating than that worn by entrapped victims in winter. However, this clothing was deliberately chosen in order to achieve the highest possible heat loss in the volunteers. This might also reflect reality to the same degree because the clothing can be destroyed in the course of traffic accidents, is removed by the rescue service personnel for medical treatment before the fire service arrives, or because of the heater in the car passengers will wear only thin clothes.

Another limitation of the study is that the floodlight may often not be directed directly to the entrapped victim as in our volunteer study, thus the poor effect in this study is overestimated compared to real life settings. Furthermore, it is possible, that existing cardiovascular diseases of our volunteers may have had the same influence on the results of the study. We excluded persons with cardiovascular medications to reduce this possible influence. In addition, by choosing a cross over design of the warming methods the potential influence if cardiovascular diseases on our results were further reduced.

Another possible limitation of the study is that we did not exclude underweight volunteers with a body mass index below 18.5 kg/m². In fact, we had only one volunteer with a body mass index below 18.5 kg/m². This may have biased our results. However, in using a crossover design between the study arms, the influence should be small.

Pathophysiological consequences on the patient’s heat balance due to severe injuries, shock, traumatic brain injury and the invasive measures required for medical care (analgesia/anaesthesia [7]), could also not be simulated in a healthy subject collective. Therefore, a drop in core body temperature is to be expected in patients in contrast to the subjects studied here [2]. Furthermore, of course, no clinical difference in outcome can be proven in a volunteer study.

## Conclusion

In the volunteer studies, it could be shown that the attempt to keep entrapped victims warm with halogen radiators does not work sufficiently. In contrast, clinically used forced air warmers work reliably even under cold ambient temperatures and are highly effective in warming subjects. It was also shown that under the used standardised experimental setting these devices are clearly superior to the passive warming method using a disposable fleece blanket. However, by using a disposable fleece blanket, heat loss can be stopped, at least in healthy subjects. From our point of view the widely used woollen blanket is an acceptable alternative for fleece that was used in our study and our finding will motivate its use in technical rescue.

### Supplementary Information

**Additional file 1.**

**Additional file 2.**

## Data Availability

The datasets used and/or analysed during the current study are available from the corresponding author on reasonable request.

## Abbreviations

ASA: American Society of Anesthesiologists Physical Status Classification System
BMI: Body mass index
BSA: Bedside shivering assessment scale
CBT: Core body temperature
DRKS: Deutsches Register klinischer Studien (German Clinical Trials registry)
MBT: Mean body temperature
MST: Mean skin temperature
NRS: Numerical rating scale
TBH: Total body heat
