# Supplementary material for: What is the best way to keep the patient warm during technical rescue? Results from two prospective randomised controlled studies with healthy volunteers
Source: BMC Emerg Med. 2023 Aug 4;23:83. doi: 10.1186/s12873-023-00850-6 (PMC10401780; doi:10.1186/s12873-023-00850-6)
Supplement: Supplementary file 1 — Additional file 1. [file 12873_2023_850_MOESM1_ESM.docx]

**QUESTIONNAIRE ACCIDENTAL HYPOTHERMIA**

Please complete the questionnaire to the best of your knowledge and return it to us in full (prepaid envelope enclosed). Please indicate a contact person for any queries in the field provided.

Thank you very much for your cooperation!

1. Name of the fire brigade:

……….………………………………………..

1. Organisation of the fire brigade:
   1. Professional fire brigade
   2. Volunteer fire brigade with full-time staff
   3. Volunteer fire brigade
   4. Plant/works fire brigade
   5. Compulsory fire brigade
2. Does your fire brigade have special structures for the medical care of patients?
   1. Yes, our fire brigade participates in the regular rescue service.
   2. Yes, namely via a first responder.
   3. Yes, through a group of comrades trained in advanced first aid.
   4. Yes, by comrades with medical training (Ambulanceman, emergency technician, paramedic etc.)
   5. No
3. Vehicles for technical assistance:
   1. Instant response vehicles for technical rescue (VRW)
   2. Dual-purpose ladder (HLF/LHF)
   3. Fire rescue unit (RW1)
   4. Heavy fire rescue unit (RW2)
   5. Equipment Truck (GW)
   6. Other : …………………
4. What means do you usually use to protect patients from cooling down?
   1. Transparent foils (to protect against glass splinters)
   2. Rescue blankets (gold/silver)
   3. Rescue blankets and work lights
   4. Wool blankets
   5. Woollen blankets and work lights
   6. Blankets and radiant heaters
   7. Active thermal ceilings (convective thermal systems)
   8. other: .........................................................................
5. When do you pay special attention to protecting patients from hypothermia?
   1. In any traffic accident
   2. Only in cold weather / at night
   3. Only occasionally, if a lot of time passes before the injured person is rescued.
   4. Not at all so far
6. How high do you estimate the risk to the patient from hypothermia?

1 - 2 - 3- 4 - 5 - 6 - 7 - 8 - 9 - 10

1. =very low risk 10 = life-threatening
2. Does the issue of patient hypothermia feed into your ongoing training?
   1. Yes, as a separate topic in the training plan
   2. Yes, in the thematic block technical assistance
   3. Yes, in the topic block First Aid
   4. No, not yet
3. In your opinion, by how many degrees does the core body temperature (CTC) of a trapped injured person, with previous normal CTC (37°C), decrease within half an hour without any warming measures? (Cool ambient temperature of 4°C, light wind, normal clothing)
   1. 0 – 0.5 °C
   2. 0.5 - 1°C
   3. 1 - 1,5°C
   4. 1.5 - 2°C
   5. more than 2°C
