# Supplementary material for: What is the best way to keep the patient warm during technical rescue? Results from two prospective randomised controlled studies with healthy volunteers
Source: BMC Emerg Med. 2023 Aug 4;23:83. doi: 10.1186/s12873-023-00850-6 (PMC10401780; doi:10.1186/s12873-023-00850-6)
Supplement: Supplementary file 2 — Additional file 2. [file 12873_2023_850_MOESM2_ESM.docx]

**Supplementary material - statistical analysis**

All numbers presented in the following are presented up to three significant digits.

**Study A**

**Body core temperature**

**Supplementary Table 1** Estimated pairwise contrasts in body core temperature between forced air warming and warming by halogen floodlight according to linear mixed effects modelling by time. Estimates are for the expected mean difference of forced air warming − halogen floodlight warming. Abbreviation: CL – limit of confidence interval.

| **Time [min]** | **Estimate [°C]** | **Standard Error** | **Lower CL** | **Upper CL** | **p.value** |
| --- | --- | --- | --- | --- | --- |
| 0 | –0.083 | 0.088 | –0.256 | 0.090 | 0.344 |
| 3 | –0.077 | 0.088 | –0.250 | 0.096 | 0.384 |
| 6 | –0.093 | 0.088 | –0.266 | 0.080 | 0.290 |
| 9 | –0.073 | 0.088 | –0.246 | 0.100 | 0.405 |
| 12 | –0.073 | 0.088 | –0.246 | 0.100 | 0.405 |
| 15 | –0.107 | 0.088 | –0.280 | 0.066 | 0.226 |
| 18 | –0.110 | 0.088 | –0.283 | 0.063 | 0.212 |
| 21 | –0.127 | 0.088 | –0.300 | 0.046 | 0.151 |
| 24 | –0.153 | 0.088 | –0.326 | 0.020 | 0.082 |
| 27 | –0.110 | 0.088 | –0.283 | 0.063 | 0.212 |
| 30 | –0.113 | 0.088 | –0.286 | 0.060 | 0.199 |
| 33 | –0.060 | 0.088 | –0.233 | 0.113 | 0.496 |
| 36 | –0.037 | 0.088 | –0.210 | 0.136 | 0.677 |
| 39 | –0.007 | 0.088 | –0.180 | 0.166 | 0.940 |
| 42 | 0.010 | 0.088 | –0.163 | 0.183 | 0.910 |
| 45 | 0.063 | 0.088 | –0.110 | 0.236 | 0.472 |
| 48 | 0.067 | 0.088 | –0.106 | 0.240 | 0.449 |
| 51 | 0.093 | 0.088 | –0.080 | 0.266 | 0.290 |
| 54 | 0.117 | 0.088 | –0.056 | 0.290 | 0.186 |
| 57 | 0.127 | 0.088 | –0.046 | 0.300 | 0.151 |
| 60 | 0.130 | 0.088 | –0.043 | 0.303 | 0.140 |

**Mean skin temperature**

**Supplementary Table 2** Estimated pairwise contrasts in mean skin temperature between forced air warming and warming by halogen floodlight according to linear mixed effects modelling by time. Estimates are for the expected mean difference of forced air warming − halogen floodlight warming. Abbreviation: CL – limit of confidence interval.

| **Time [min]** | **Estimate [°C]** | **Standard Error** | **Lower CL** | **Upper CL** | **p.value** |
| --- | --- | --- | --- | --- | --- |
| 0 | –0.131 | 0.555 | –1.220 | 0.957 | 0.813 |
| 3 | –0.100 | 0.555 | –1.188 | 0.989 | 0.857 |
| 6 | 0.120 | 0.555 | –0.969 | 1.208 | 0.829 |
| 9 | –0.102 | 0.555 | –1.191 | 0.987 | 0.854 |
| 12 | –0.231 | 0.555 | –1.319 | 0.858 | 0.678 |
| 15 | 0.080 | 0.555 | –1.009 | 1.168 | 0.886 |
| 18 | –0.061 | 0.555 | –1.150 | 1.027 | 0.912 |
| 21 | –0.260 | 0.555 | –1.349 | 0.829 | 0.639 |
| 24 | 0.721 | 0.555 | –0.367 | 1.810 | 0.194 |
| 27 | 2.800 | 0.555 | 1.711 | 3.889 | 0.000 |
| 30 | 4.721 | 0.555 | 3.632 | 5.809 | 0.000 |
| 33 | 6.487 | 0.555 | 5.398 | 7.576 | 0.000 |
| 36 | 7.323 | 0.555 | 6.235 | 8.412 | 0.000 |
| 39 | 7.611 | 0.555 | 6.522 | 8.699 | 0.000 |
| 42 | 7.938 | 0.555 | 6.849 | 9.027 | 0.000 |
| 45 | 8.099 | 0.555 | 7.010 | 9.188 | 0.000 |
| 48 | 7.897 | 0.555 | 6.809 | 8.986 | 0.000 |
| 51 | 8.285 | 0.555 | 7.196 | 9.374 | 0.000 |
| 54 | 8.397 | 0.555 | 7.308 | 9.485 | 0.000 |
| 57 | 9.098 | 0.555 | 8.010 | 10.187 | 0.000 |
| 60 | 9.032 | 0.555 | 7.943 | 10.120 | 0.000 |

**Mean body temperature**

**Supplementary Table 3** Estimated pairwise contrasts in mean body temperature between forced air warming and warming by halogen floodlight according to linear mixed effects modelling by time. Estimates are for the expected mean difference of forced air warming − halogen floodlight warming. Abbreviation: CL – limit of confidence interval.

| **Time [min]** | **Estimate [°C]** | **Standard Error** | **Lower CL** | **Upper CL** | **p.value** |
| --- | --- | --- | --- | --- | --- |
| 0 | –0.101 | 0.206 | –0.505 | 0.304 | 0.625 |
| 3 | –0.085 | 0.206 | –0.489 | 0.319 | 0.680 |
| 6 | –0.017 | 0.206 | –0.421 | 0.387 | 0.936 |
| 9 | –0.084 | 0.206 | –0.488 | 0.320 | 0.685 |
| 12 | –0.130 | 0.206 | –0.534 | 0.274 | 0.528 |
| 15 | –0.040 | 0.206 | –0.444 | 0.365 | 0.848 |
| 18 | –0.161 | 0.206 | –0.565 | 0.244 | 0.436 |
| 21 | –0.175 | 0.206 | –0.579 | 0.229 | 0.397 |
| 24 | 0.162 | 0.206 | –0.243 | 0.566 | 0.433 |
| 27 | 0.938 | 0.206 | 0.533 | 1.342 | 0.000 |
| 30 | 1.627 | 0.206 | 1.223 | 2.031 | 0.000 |
| 33 | 2.297 | 0.206 | 1.893 | 2.701 | 0.000 |
| 36 | 2.613 | 0.206 | 2.209 | 3.017 | 0.000 |
| 39 | 2.736 | 0.206 | 2.331 | 3.140 | 0.000 |
| 42 | 2.864 | 0.206 | 2.460 | 3.268 | 0.000 |
| 45 | 2.956 | 0.206 | 2.552 | 3.360 | 0.000 |
| 48 | 2.886 | 0.206 | 2.482 | 3.290 | 0.000 |
| 51 | 3.042 | 0.206 | 2.638 | 3.446 | 0.000 |
| 54 | 3.097 | 0.206 | 2.693 | 3.502 | 0.000 |
| 57 | 3.356 | 0.206 | 2.952 | 3.761 | 0.000 |
| 60 | 3.335 | 0.206 | 2.930 | 3.739 | 0.000 |

**Total body heat**

**Supplementary Table 4** Estimated pairwise contrasts in total body heat between forced air warming and warming by halogen floodlight according to linear mixed effects modelling by time. Estimates are for the expected mean difference of forced air warming − halogen floodlight warming. Abbreviation: CL – limit of confidence interval.

| **Time [min]** | **Estimate [J]** | **Standard Error** | **Lower CL** | **Upper CL** | **p.value** |
| --- | --- | --- | --- | --- | --- |
| 0 | –48.173 | 59.7 | –165.303 | 68.956 | 0.420 |
| 3 | –45.105 | 59.7 | –162.234 | 72.025 | 0.450 |
| 6 | –29.987 | 59.7 | –147.117 | 87.143 | 0.616 |
| 9 | –41.214 | 59.7 | –158.344 | 75.915 | 0.490 |
| 12 | –51.011 | 59.7 | –168.141 | 66.119 | 0.393 |
| 15 | –28.249 | 59.7 | –145.379 | 88.881 | 0.636 |
| 18 | –73.231 | 59.7 | –190.361 | 43.898 | 0.220 |
| 21 | –65.628 | 59.7 | –182.757 | 51.502 | 0.272 |
| 24 | 25.478 | 59.7 | –91.652 | 142.607 | 0.670 |
| 27 | 251.473 | 59.7 | 134.343 | 368.603 | 0.000 |
| 30 | 437.060 | 59.7 | 319.930 | 554.190 | 0.000 |
| 33 | 625.440 | 59.7 | 508.310 | 742.570 | 0.000 |
| 36 | 708.262 | 59.7 | 591.132 | 825.392 | 0.000 |
| 39 | 742.369 | 59.7 | 625.239 | 859.499 | 0.000 |
| 42 | 786.552 | 59.7 | 669.423 | 903.682 | 0.000 |
| 45 | 810.036 | 59.7 | 692.906 | 927.166 | 0.000 |
| 48 | 803.838 | 59.7 | 686.708 | 920.968 | 0.000 |
| 51 | 844.916 | 59.7 | 727.787 | 962.046 | 0.000 |
| 54 | 862.188 | 59.7 | 745.059 | 979.318 | 0.000 |
| 57 | 940.297 | 59.7 | 823.167 | 1057.427 | 0.000 |
| 60 | 934.282 | 59.7 | 817.153 | 1051.412 | 0.000 |

**NRS Score**

**Supplementary Table 5** Estimated pairwise contrasts in NRS score between forced air warming and warming by halogen floodlight according to linear mixed effects modelling by time. Estimates are for the expected mean difference of forced air warming − halogen floodlight warming. Abbreviation: CL – limit of confidence interval.

| **Time [min]** | **Estimate [points]** | **Standard Error** | **Lower CL** | **Upper CL** | **p.value** |
| --- | --- | --- | --- | --- | --- |
| 0 | 0.000 | 0.278 | –0.546 | 0.546 | 1.000 |
| 3 | 0.000 | 0.278 | –0.546 | 0.546 | 1.000 |
| 6 | 0.133 | 0.278 | –0.412 | 0.679 | 0.632 |
| 9 | 0.067 | 0.278 | –0.479 | 0.612 | 0.811 |
| 12 | 0.033 | 0.278 | –0.512 | 0.579 | 0.905 |
| 15 | 0.100 | 0.278 | –0.446 | 0.646 | 0.719 |
| 18 | –0.167 | 0.278 | –0.712 | 0.379 | 0.549 |
| 21 | 0.033 | 0.278 | –0.512 | 0.579 | 0.905 |
| 24 | –1.167 | 0.278 | –1.712 | –0.621 | 0.000 |
| 27 | –2.967 | 0.278 | –3.512 | –2.421 | 0.000 |
| 30 | –3.833 | 0.278 | –4.379 | –3.288 | 0.000 |
| 33 | –4.267 | 0.278 | –4.812 | –3.721 | 0.000 |
| 36 | –4.600 | 0.278 | –5.146 | –4.054 | 0.000 |
| 39 | –4.767 | 0.278 | –5.312 | –4.221 | 0.000 |
| 42 | –4.933 | 0.278 | –5.479 | –4.388 | 0.000 |
| 45 | –5.067 | 0.278 | –5.612 | –4.521 | 0.000 |
| 48 | –5.300 | 0.278 | –5.846 | –4.754 | 0.000 |
| 51 | –5.567 | 0.278 | –6.112 | –5.021 | 0.000 |
| 54 | –5.733 | 0.278 | –6.279 | –5.188 | 0.000 |
| 57 | –5.800 | 0.278 | –6.346 | –5.254 | 0.000 |
| 60 | –5.900 | 0.278 | –6.446 | –5.354 | 0.000 |

**Study B**

**Body core temperature**

**Supplementary Table 6** Estimated pairwise contrasts in body core temperature between forced air warming and warming using a fleece blanket according to linear mixed effects modelling by time. Estimates are for the expected mean difference of forced air warming − fleece blanket warming. Abbreviation: CL – limit of confidence interval.

| **Time [min]** | **Estimate [°C]** | **Standard Error** | **Lower CL** | **Upper CL** | **p.value** |
| --- | --- | --- | --- | --- | --- |
| 0 | 0.012 | 0.074 | –0.133 | 0.158 | 0.866 |
| 3 | 0.016 | 0.074 | –0.129 | 0.161 | 0.833 |
| 6 | –0.028 | 0.074 | –0.173 | 0.117 | 0.704 |
| 9 | 0.009 | 0.074 | –0.136 | 0.154 | 0.899 |
| 12 | 0.019 | 0.074 | –0.126 | 0.164 | 0.800 |
| 15 | –0.003 | 0.074 | –0.148 | 0.142 | 0.966 |
| 18 | –0.003 | 0.074 | –0.148 | 0.142 | 0.966 |
| 21 | 0.003 | 0.074 | –0.142 | 0.148 | 0.966 |
| 24 | 0.000 | 0.074 | –0.145 | 0.145 | 1.000 |
| 27 | 0.031 | 0.074 | –0.114 | 0.176 | 0.673 |
| 30 | –0.009 | 0.074 | –0.154 | 0.136 | 0.899 |
| 33 | –0.006 | 0.074 | –0.151 | 0.139 | 0.933 |
| 36 | –0.003 | 0.074 | –0.148 | 0.142 | 0.966 |
| 39 | –0.016 | 0.074 | –0.161 | 0.129 | 0.833 |
| 42 | –0.013 | 0.074 | –0.158 | 0.133 | 0.866 |
| 45 | –0.009 | 0.074 | –0.154 | 0.136 | 0.899 |
| 48 | –0.009 | 0.074 | –0.154 | 0.136 | 0.899 |
| 51 | 0.000 | 0.074 | –0.145 | 0.145 | 1.000 |
| 54 | 0.006 | 0.074 | –0.139 | 0.151 | 0.933 |
| 57 | –0.003 | 0.074 | –0.148 | 0.142 | 0.966 |
| 60 | 0.003 | 0.074 | –0.142 | 0.148 | 0.966 |

**Mean skin temperature**

**Supplementary Table 7** Estimated pairwise contrasts in mean skin temperature between forced air warming and warming using a fleece blanket according to linear mixed effects modelling by time. Estimates are for the expected mean difference of forced air warming − fleece blanket warming. Abbreviation: CL – limit of confidence interval.

| **Time [min]** | **Estimate [°C]** | **Standard Error** | **Lower CL** | **Upper CL** | **p.value** |
| --- | --- | --- | --- | --- | --- |
| 0 | 0.155 | 0.279 | –0.393 | 0.702 | 0.579 |
| 3 | 0.143 | 0.279 | –0.405 | 0.690 | 0.610 |
| 6 | 0.130 | 0.279 | –0.417 | 0.677 | 0.641 |
| 9 | 0.295 | 0.279 | –0.252 | 0.843 | 0.290 |
| 12 | 0.194 | 0.279 | –0.354 | 0.741 | 0.488 |
| 15 | –0.153 | 0.279 | –0.701 | 0.394 | 0.582 |
| 18 | 0.022 | 0.279 | –0.525 | 0.570 | 0.936 |
| 21 | 0.408 | 0.279 | –0.139 | 0.956 | 0.143 |
| 24 | 0.841 | 0.279 | 0.293 | 1.388 | 0.003 |
| 27 | 1.222 | 0.279 | 0.675 | 1.770 | 0.000 |
| 30 | 1.528 | 0.279 | 0.980 | 2.075 | 0.000 |
| 33 | 1.735 | 0.279 | 1.188 | 2.283 | 0.000 |
| 36 | 1.934 | 0.279 | 1.386 | 2.481 | 0.000 |
| 39 | 2.336 | 0.279 | 1.789 | 2.884 | 0.000 |
| 42 | 2.188 | 0.279 | 1.641 | 2.736 | 0.000 |
| 45 | 2.351 | 0.279 | 1.804 | 2.899 | 0.000 |
| 48 | 2.436 | 0.279 | 1.889 | 2.984 | 0.000 |
| 51 | 2.572 | 0.279 | 2.025 | 3.120 | 0.000 |
| 54 | 2.438 | 0.279 | 1.891 | 2.986 | 0.000 |
| 57 | 2.498 | 0.279 | 1.950 | 3.045 | 0.000 |
| 60 | 2.420 | 0.279 | 1.872 | 2.967 | 0.000 |

**Mean body temperature**

**Supplementary Table 8** Estimated pairwise contrasts in mean body temperature between forced air warming and warming using a fleece blanket according to linear mixed effects modelling by time. Estimates are for the expected mean difference of forced air warming − fleece blanket warming. Abbreviation: CL – limit of confidence interval.

| **Time [min]** | **Estimate [°C]** | **Standard Error** | **Lower CL** | **Upper CL** | **p.value** |
| --- | --- | --- | --- | --- | --- |
| 0 | 0.064 | 0.113 | –0.158 | 0.286 | 0.573 |
| 3 | 0.061 | 0.113 | –0.161 | 0.283 | 0.588 |
| 6 | 0.029 | 0.113 | –0.193 | 0.251 | 0.799 |
| 9 | 0.112 | 0.113 | –0.110 | 0.334 | 0.321 |
| 12 | 0.082 | 0.113 | –0.140 | 0.304 | 0.470 |
| 15 | –0.057 | 0.113 | –0.279 | 0.165 | 0.613 |
| 18 | 0.006 | 0.113 | –0.216 | 0.228 | 0.957 |
| 21 | 0.141 | 0.113 | –0.081 | 0.363 | 0.212 |
| 24 | 0.303 | 0.113 | 0.081 | 0.524 | 0.008 |
| 27 | 0.460 | 0.113 | 0.238 | 0.682 | 0.000 |
| 30 | 0.544 | 0.113 | 0.322 | 0.766 | 0.000 |
| 33 | 0.621 | 0.113 | 0.399 | 0.843 | 0.000 |
| 36 | 0.694 | 0.113 | 0.472 | 0.916 | 0.000 |
| 39 | 0.831 | 0.113 | 0.609 | 1.053 | 0.000 |
| 42 | 0.780 | 0.113 | 0.558 | 1.002 | 0.000 |
| 45 | 0.840 | 0.113 | 0.619 | 1.062 | 0.000 |
| 48 | 0.871 | 0.113 | 0.649 | 1.093 | 0.000 |
| 51 | 0.926 | 0.113 | 0.704 | 1.148 | 0.000 |
| 54 | 0.882 | 0.113 | 0.660 | 1.104 | 0.000 |
| 57 | 0.897 | 0.113 | 0.675 | 1.119 | 0.000 |
| 60 | 0.873 | 0.113 | 0.651 | 1.095 | 0.000 |

**Total body heat**

**Supplementary Table 9** Estimated pairwise contrasts in total body heat between forced air warming and warming using a fleece blanket according to linear mixed effects modelling by time. Estimates are for the expected mean difference of forced air warming − fleece blanket warming. Abbreviation: CL – limit of confidence interval.

| **Time [min]** | **Estimate [J]** | **Standard Error** | **Lower CL** | **Upper CL** | **p.value** |
| --- | --- | --- | --- | --- | --- |
| 0 | 19.632 | 33.267 | –45.631 | 84.896 | 0.555 |
| 3 | 20.930 | 33.267 | –44.334 | 86.193 | 0.529 |
| 6 | 9.191 | 33.267 | –56.073 | 74.455 | 0.782 |
| 9 | 29.485 | 33.267 | –35.779 | 94.749 | 0.376 |
| 12 | 26.103 | 33.267 | –39.160 | 91.367 | 0.433 |
| 15 | –15.478 | 33.267 | –80.742 | 49.785 | 0.642 |
| 18 | 2.274 | 33.267 | –62.990 | 67.537 | 0.946 |
| 21 | 35.256 | 33.267 | –30.008 | 100.519 | 0.289 |
| 24 | 81.424 | 33.267 | 16.160 | 146.687 | 0.015 |
| 27 | 125.005 | 33.267 | 59.742 | 190.269 | 0.000 |
| 30 | 141.757 | 33.267 | 76.493 | 207.021 | 0.000 |
| 33 | 162.274 | 33.267 | 97.010 | 227.537 | 0.000 |
| 36 | 187.216 | 33.267 | 121.952 | 252.480 | 0.000 |
| 39 | 226.814 | 33.267 | 161.551 | 292.078 | 0.000 |
| 42 | 205.573 | 33.267 | 140.309 | 270.836 | 0.000 |
| 45 | 231.730 | 33.267 | 166.466 | 296.993 | 0.000 |
| 48 | 237.856 | 33.267 | 172.593 | 303.120 | 0.000 |
| 51 | 249.353 | 33.267 | 184.089 | 314.617 | 0.000 |
| 54 | 239.612 | 33.267 | 174.348 | 304.875 | 0.000 |
| 57 | 244.765 | 33.267 | 179.501 | 310.028 | 0.000 |
| 60 | 243.972 | 33.267 | 178.709 | 309.236 | 0.000 |

**NRS Score**

**Supplementary Table 10** Estimated pairwise contrasts in NRS score between forced air warming and warming using a fleece blanket according to linear mixed effects modelling by time. Estimates are for the expected mean difference of forced air warming − fleece blanket warming. Abbreviation: CL – limit of confidence interval.

| **Time [min]** | **Estimate [points]** | **Standard Error** | **Lower CL** | **Upper CL** | **p.value** |
| --- | --- | --- | --- | --- | --- |
| 0 | 0.125 | 0.274 | –0.412 | 0.662 | 0.648 |
| 3 | 0.219 | 0.274 | –0.318 | 0.755 | 0.424 |
| 6 | 0.406 | 0.274 | –0.130 | 0.943 | 0.138 |
| 9 | 0.344 | 0.274 | –0.193 | 0.880 | 0.209 |
| 12 | 0.281 | 0.274 | –0.255 | 0.818 | 0.304 |
| 15 | 0.063 | 0.274 | –0.474 | 0.599 | 0.819 |
| 18 | –0.063 | 0.274 | –0.599 | 0.474 | 0.819 |
| 21 | 0.000 | 0.274 | –0.537 | 0.537 | 1.000 |
| 24 | –1.312 | 0.274 | –1.849 | –0.776 | 0.000 |
| 27 | –1.625 | 0.274 | –2.162 | –1.088 | 0.000 |
| 30 | –1.688 | 0.274 | –2.224 | –1.151 | 0.000 |
| 33 | –1.688 | 0.274 | –2.224 | –1.151 | 0.000 |
| 36 | –2.000 | 0.274 | –2.537 | –1.463 | 0.000 |
| 39 | –2.094 | 0.274 | –2.630 | –1.557 | 0.000 |
| 42 | –2.187 | 0.274 | –2.724 | –1.651 | 0.000 |
| 45 | –2.406 | 0.274 | –2.943 | –1.870 | 0.000 |
| 48 | –2.594 | 0.274 | –3.130 | –2.057 | 0.000 |
| 51 | –2.719 | 0.274 | –3.255 | –2.182 | 0.000 |
| 54 | –2.844 | 0.274 | –3.380 | –2.307 | 0.000 |
| 57 | –2.969 | 0.274 | –3.505 | –2.432 | 0.000 |
| 60 | –3.000 | 0.274 | –3.537 | –2.463 | 0.000 |
